# Knowledge, Attitudes, and Practices Regarding Zika: Paper- and Internet-Based Survey in Zhejiang, China

**DOI:** 10.2196/publichealth.7663

**Published:** 2017-10-30

**Authors:** Yu Huang, Shuiyang Xu, Lei Wang, Yushui Zhao, He Liu, Dingming Yao, Yue Xu, Qiaohong Lv, Gang Hao, Yan Xu, Qingqing Wu

**Affiliations:** ^1^ Department of Health Education Zhejiang Provincial Center for Disease Control and Prevention Hangzhou China

**Keywords:** Zika virus disease, knowledge, attitudes, and practice, Internet-based survey, paper-based survey

## Abstract

**Background:**

As public access to the Internet increases, many health workers prefer to carry out health education online, reducing the use of traditional community-based health education methods. Since March 2016, four Zika cases have been confirmed in Zhejiang, China. Rapid assessment of people’s knowledge, attitudes, and practices (KAP) regarding Zika is crucial to its prevention and control. Web-based surveys to assess public KAP may be a growing trend; however, we had little experience with this method.

**Objectives:**

The aim of this study was to explore KAP regarding Zika in residents of Zhejiang using both traditional paper- and innovative Internet-based investigations.

**Methods:**

A questionnaire was designed by Zhejiang Provincial Center for Disease Control and Prevention. A paper-based version of the survey was used in a cross-sectional community study following multistage cluster random sampling, and an Internet-based survey was promoted through a local health education site. Data were interpreted via univariate and multivariate analyses.

**Results:**

A total of 447 community residents participated in the paper-based survey, with a response rate of 89.4% (447/500), and 621 eligible Internet users participated in the Internet-based survey, with a response rate of 36.92% (621/1682). Age, education level, and occupation differed significantly between participants in the paper- and Internet-based surveys. Participants completing the Internet-based survey were much younger (χ^2^_2_=144.7, *P*<.001) and had a higher level of education (χ^2^_2_=423.5, *P*<.001) than those completing the paper-based survey. Among participants completing the paper-based survey, there were more farmers, housewives, and unemployed people (χ^2^_3_=413.7, *P*<.001). Overall, 83.52% of participants (892/1068) knew the transmission route for Zika, 76.12% (813/1068) knew that pregnant women were at high risk of severe complications, 66.39% (709/1068) knew that contracting Zika during pregnancy could lead to newborn babies with microcephaly, and 98.88% (1056/1068) knew places where mosquitos could usually be found. After controlling for sociodemographic variables, participants completing the Internet-based survey were more likely to know the transmission route of Zika (odds ratio [OR]=5.0, 95% CI 3.0-8.0), the association between pregnant women with Zika and newborn babies with microcephaly (OR 2.1, 95% CI 1.4-3.0), and that pregnant women were at high risk for Zika (OR 5.5, 95% CI 3.5-8.4) than those completing the paper-based survey. They were less likely to worry about contracting Zika (OR 0.6, 95% CI 0.4-0.9) and more likely to actively seek information about Zika than participants completing the paper-based survey (OR 3.3, 95% CI 2.0-5.6).

**Conclusions:**

Participants completing the Internet-based survey had a higher level of basic knowledge and more positive attitudes and behaviors than participants completing the paper-based survey. In addition to providing Web-based health information, the government should ensure sufficient access to health information for the elderly and less educated people in the community to improve health equity.

## Introduction

Zika is a mosquito-borne flavivirus that was first identified in a rhesus monkey in the Zika Forest of Uganda in 1947 [1]. Such as the dengue virus, the symptoms of the Zika virus tend to be mild. Symptoms of Zika include fever, rash, pain in the joints, and pink eye [2]. Zika is transmitted by the common mosquito vector, *Aedes* species. In 2015, the international spread of Zika infection attracted global attention, when a marked increase in microcephaly cases in Brazil was linked to an epidemic of Zika infection [3].

In 2016, there were 24 imported Zika cases in China. Zhejiang province, located in southeast China and famous worldwide for its small commodity trade and vibrant market, had four of these Zika cases imported from South America [4]. Zhejiang exports 65% of its products to over 215 countries and regions, especially Southeast Asia and South America where Zika is endemic [5]. Moreover, Zhejiang has a hot, humid climate in summer, making it a suitable habitat for the *Aedes* species [6].

With the emergence of a new disease, inaccurate information and negative attitudes may lead to unnecessary concerns, rumors, and even panic [7,8]. Therefore, it is important for governments and health workers to assess the public’s knowledge, attitudes, and practices (KAP) toward Zika. Traditionally, paper-based questionnaires have been used for research such as this. However, these take time, energy, and resources to administer. As public access increases, the Internet offers an unprecedented opportunity to conduct rapid assessments of community KAP related to emerging new diseases. Internet-based investigations are cost-effective, have rapid data collection, and the data can easily be transferred to statistical software for analysis. Some traditional paper-based investigations have already been superseded by Internet-based ones [9,10]. A disadvantage of Internet-based investigations is that data collected online may not be representative of the general population. However, data collected in the paper-based investigation may not be representative either. Web-based surveys are considered more feasible for large population-based cross-sectional studies [11].

The speed of data collection with Web-based surveys far exceeds that of paper-based investigations, which is what is needed for emergency health education and risk communication. Some research on infectious diseases is already based on Web-based data [12,13]. However, we found no study that compared Internet- and paper-based surveys in Zhejiang regarding emerging infectious diseases such as Zika.

The purpose of this study was to explore KAP regarding Zika in Zhejiang by using both paper- and Internet-based investigations. We also wanted to compare a traditional paper-based investigation with an innovative Internet-based investigation, providing scientific evidence for future assessments of emergency health education needs.

## Methods

### Questionnaire

The questionnaire was designed by the Zhejiang Provincial Center for Disease Control and Prevention (Zhejiang CDC). It collected sociodemographic data (sex, age, occupation, education level, and residential area); KAP regarding Zika; and sources of information on Zika. The questions about knowledge of Zika were (1) How is Zika transmitted to humans? (2) Which population group is at a high risk of severe complications from Zika? (3) If pregnant women contract Zika, what is the adverse outcome? and (4) Where are mosquitoes extremely common? Questions about attitudes to Zika were (1) Do you worry about contracting Zika? and (2) How do you think your body will be harmed if you contract Zika? Questions about practices related to Zika were (1) Do you use strategies to prevent mosquito bites at home? and (2) Do you actively seek information regarding Zika? Questions about Zika were based on the latest official report of World Health Organization and literature. The original questionnaire was developed in Chinese by research group members and refined over two rounds of Delphi method collaboration.

### Recruitment

Participants of the Internet-based survey were recruited via Zhejiang Health Education, a public WeChat site with 100,000 users in the Zhejiang Province of China that is used to popularize health knowledge. A message stating “A 10-minute investigation on Zika will get you 20 RMB cell phone recharge” was created on the Zhejiang Health Education site, with a link to the questionnaire. Users who viewed the message could share it through the Internet, which allows news and information to be spread quickly to a large number of people. The questionnaire was hosted on *wenjuan* a large free questionnaire platform that has an innovative editing interface and a result analysis interface.

Participants who completed the paper-based survey were recruited from among community residents at Yiwu and Hangzhou, the two cities in Zhejiang Province where the first four Zika cases were found. Hangzhou is the capital of Zhejiang Province, and Yiwu is a prefecture city. Multistage cluster random sampling was undertaken. First, one district was selected randomly from each city. Then, one community was randomly selected from each of these districts. Families in the chosen communities were numbered and again chosen randomly. Finally, the randomly selected family members were informed about the study by a leaflet that contained the same information provided online and were invited to complete the questionnaire. Contact with potential participants was made by local health center professionals conducting a home visit. For each questionnaire, an investigator received 25 RMB and a respondent received a gift worth 20 RMB. We assumed that, overall, the level of those in the community with good knowledge about Zika would be about 50%, with 95% confidence in the estimate, precision of 5%, and 20% of nonresponse rate. Hence, the minimum required sample size was 500 participants.

The study was approved by the ethics committee at Zhejiang CDC. Informed consent was obtained from all participants before their information was collected.

### Statistical Analysis

Data from paper-based surveys were entered onto EpiData 3.03 (EpiData Association). Internet-based data were exported from the wenjuan to Excel (Microsoft). Data were analyzed using Statistical Package for the Social Sciences (SPSS) version 19.0 (IBM Corp). Standard descriptive statistics were used to summarize the data. Demographic characteristics of participants taking the Internet- and paper-based surveys were compared using chi-square tests. Chi-square tests were also used to explore differences in KAP regarding Zika between the two groups. Univariate and multivariate analyses were conducted to explore the association between sociodemographic variables and KAP variables. Items of KAP were analyzed separately. We dummy-coded categorical variables. Participants who were able to correctly answer the knowledge-based questions about transmission of Zika, main at-risk population, adverse outcomes of Zika during pregnancy, and where mosquitoes were extremely common were coded into the high-KAP category; all others were coded into the low-KAP category. Participants who were not at all worried about contracting Zika were coded into one category; the remainder were coded into another category. Those who replied that contracting Zika could cause serious harm were coded into one category; all others were coded into another category. For practices related to Zika, participants who carried out strategies to prevent mosquitoes and those who actively sought information about Zika were coded into one category; all others were coded into another category. *P* values of <.05 were considered statistically significant (two-sided).

## Results

### Differences in Sociodemographic Characteristics Between Participants of Paper- and Internet-Based Surveys

A total of 1682 WeChat users from Zhejiang Province visited the page hosting the Web-based survey, and 36.92% (621/1682) of them completed the questionnaire and were included in the study. Of the 500 community residents in Zhejiang Province who were invited to participate in the paper-based survey, 53 refused and 447 agreed. The response rate was 89.4% (447/500).

The proportion of participants aged <30, 30-49, and ≥50 years was 17.0% (76/447), 50.6% (226/447), and 32.4% (145/447), respectively, among those completing the paper-based survey and 25.1% (156/621), 70.1% (435/621), and 4.8% (30/621), respectively, among those completing the Internet-based survey. There was a significant difference between the two groups (χ^2^_2_=144.7, *P*<.001). Across all participants, 69.57% (743/1068) were female, and 30.43% (325/1068) were male, with no difference detected between those completing the Internet- and paper-based surveys. Among participants completing the Internet-based survey, 91.1% (566/621) had a postsecondary education level, which was a much higher level than participants completing the paper-based survey (χ^2^_2_=423.5, *P*<.001).

**Table 1:** Sociodemographic characteristic of participants completing Internet- and paper-based surveys.

Characteristic		Investigation approach		Total (n=1068), n (%)	χ^2^ (degrees of freedom)	*P* value
		Paper-based (N=447), n (%)	Internet-based (N=621), n (%)			
**Sex**						
	Female	305 (68.2)	438 (70.5)	743 (69.57)	0.6 (1)	.42
	Male	142 (31.8)	183 (29.5)	325 (30.43)		
**Age (years)**						
	<30	76 (17.0)	156 (25.1)	232 (21.72)	144.7 (2)	<.001
	30-49	226 (50.6)	435 (70.1)	661 (61.89)		
	≥50	145 (32.4)	30 (4.8)	175 (16.39)		
**Education**						
	Primary or less (<9 years)	194 (43.4)	15 (2.4)	209 (19.57)	423.5 (2)	<.001
	Secondary (9-12 years)	111 (24.8)	40 (6.5)	151 (14.14)		
	Postsecondary (>12 years)	142 (31.8)	566 (91.1)	708 (66.29)		
**Occupation**						
	Staff of government institution	51 (11.4)	451 (72.6)	502 (47.00)	413.7 (3)	<.001
	Staff of business or service industry	139 (31.1)	71 (11.4)	210 (19.66)		
	Farmer	69 (15.4)	3 (0.5)	72 (6.74)		
	Other (eg, retired, housewife or househusband, and unemployed)	188 (42.1)	96 (15.5)	284 (26.59)		

The proportion of participants who worked in government institutions, in businesses or service industries, as farmers, and other occupation groups (eg, retired, housewife, househusband, and unemployed) were 11.4% (51/447), 31.1% (139/447), 15.4% (69/447), and 42.1% (188/447), respectively, among those completing paper-based surveys and 72.6% (451/621), 11.4% (71/621), 0.5% (3/621), and 15.5% (96/621), respectively, among those completing Internet-based surveys. The chi-square test found a significant difference in occupation distribution between the two groups (χ^2^_3_=413.7, *P*<.001; Table 1).

### KAP Regarding Zika by Investigation Approach

#### Knowledge Regarding Zika

Among the total participants, 83.52% (892/1068) had some knowledge of the transmission route of Zika. The knowledge that the Zika virus was primarily transmitted to humans through mosquito bites was demonstrated by 95.2% (591/621) and 67.3% (301/447) of participants completing the Internet-based and paper-based surveys, respectively; a statistically significant difference (χ^2^_2_=152.1, *P*<.001). A total of 76.12% of all participants (813/1068) knew that pregnant women were at high risk of severe complications. This group was made up of 90.0% (559/621) and 56.8% (254/447) of participants completing the Internet-based and paper-based surveys, respectively; another statistically significant difference (χ^2^_2_=1173.7, *P*<.001). The association between Zika infection during pregnancy and newborn babies with microcephaly was known by 66.39% (709/1068) of all participants. This knowledge was demonstrated by 80.0% (497/621) and 47.4% (212/447) of participants completing the Internet-based and paper-based surveys, respectively; a statistically significant difference (χ^2^_2_=190.5, *P*<.001). Of the 1068 total participants, 1056 (98.88%) knew that mosquitos were extremely common in dark, wet places such as river banks and parks. This group was made up of 98.4% (611/621) and 99.5% (445/447) of participants completing the Internet-based and paper-based surveys, respectively, which was a statistically significant difference (χ^2^_2_=684.9, *P*<.001; Table 2).

#### Attitudes Regarding Zika

Of the 1068 of total participants, 502 (47.00%) were worried about contracting Zika. This group was made up of 55.6% (345/621) and 35.1% (157/447) of participants completing the Internet-based and paper-based surveys, respectively. The difference between the two groups was found to be significant (χ^2^_1_=43.6, *P*<.001). Potential harm due to contracting Zika was thought to be serious, mild, and nil by 31.39% (335/1068), 62.83% (671/1068), and 5.81% (62/1068), respectively. There was no difference between the two participant groups (χ^2^_2_=90.7, *P*=.60; Table 2).

#### Practices Regarding Zika

Among the total participants, 98.50% (1052/1068) carried out preventive strategies such as using a mosquito net or mosquito repellent. There was no significant difference in practices between participants completing the Internet-based survey and the paper-based survey (χ^2^_1_=2.8, *P*=.09). There was a significant difference between the two participant groups regarding seeking out information about Zika (χ^2^_1_=90.7, *P*<.001). Among all participants, 84.64% (904/1068) actively sought out information, made up of 93.6% (581/624) and 72.3% (323/447) of participants completing the Internet- and the paper-based surveys, respectively. (Table 2).

**Table 2:** Knowledge, attitudes, and practices regarding Zika of participants completing Internet- and paper-based surveys.

Variables			Investigation approach		Total (N=1068), n (%)	χ^2^ (degrees of freedom)	*P* value
			Paper-based (N=447), n (%)	Internet-based (N=621), n (%)			
**Knowledge**							
	**Zika virus is primarily transmitted to humans through mosquito bites**						
		Yes	301 (67.3)	591 (95.2)	892 (83.52)	152.1 (2)	<.001
		No	108 (24.2)	30 (4.8)	138 (12.92)		
		Unclear	38 (8.5)	0 (0.0)	38 (3.56)		
	**Pregnant women are at a high risk of severe complications from Zika**						
		Yes	254 (56.8)	559 (90.0)	813 (76.12)	173.7 (2)	<.001
		No	105 (23.5)	54 (8.7)	159 (14.89)		
		Unclear	88 (19.7)	8 (1.3)	96 (8.99)		
	**Pregnant women infected with might have newborn babies with microcephaly**						
		Yes	212 (47.4)	497 (80.0)	709 (66.39)	190.5 (2)	<.001
		No	93 (20.8)	107 (17.2)	200 (18.73)		
		Unclear	142 (31.8)	17 (2.7)	159 (14.89)		
	**Where are mosquitoes extremely common**						
		Dark wet places	445 (99.5)	611 (98.4)	1056 (98.88)	3.3 (2)	.19
		Other	2 (0.5)	9 (1.4)	11 (1.02)		
		Unclear	0 (0.0)	1 (0.2)	1 (0.10)		
**Attitudes**							
	**Do you worry about contracting Zika**						
		Yes	157 (35.1)	345 (55.6)	502 (47.00)	43.6 (1)	<.001
		No	290 (64.9)	276 (44.4)	566 (53.00)		
	**How much harm do you think would occur to your body if you contracted Zika**						
		Serious	147 (32.9)	188 (30.3)	335 (31.39)	1.0 (2)	.60
		Mild	273 (61.1)	398 (64.1)	671 (62.83)		
		No harm	27 (6.0)	35 (5.6)	62 (5.81)		
**Practices**							
	**Carry out strategies to prevent mosquito bites at home**						
		Yes	437 (97.8)	615 (99.0)	1052 (98.50)	2.8 (1)	.09
		No	10 (2.2)	6 (1.0)	16 (1.50)		
	**Actively seeks information about Zika**						
		Yes	323 (72.3)	581 (93.6)	904 (84.64)	90.7 (1)	<.001
		No	124 (27.7)	40 (6.4)	164 (15.36)		

**Table 3:** Unadjusted and adjusted (multivariate logistic regression, adjusted for the other factors shown in the table) odds ratios (OR) and 95% CI for knowledge about Zika by sociodemographic variables.

Predictors		Zika virus is primarily transmitted to humans through mosquito bites				Pregnant women are the main at-risk population for Zika			
		OR^a^ (95% CI)	*P* value	AOR^b^ (95% CI)	*P* value	OR (95% CI)	*P* value	AOR (95% CI)	*P* value
**Sex**									
	Female	1		1		1		1	
	Male	0.8 (0.6-1.2)	.26	0.8 (0.6-1.3)	.40	1.1 (0.8-1.4)	.71	1.0 (0.7-1.5)	.81
**Age (years)**									
	<30	1		1		1		1	
	30-49	1.2 (0.8-1.9)	.33	1.5 (0.9-2.4)	.12	1.4 (1.0-2.0)	<.001	1.6 (1.0-2.3)	.03
	≥50	0.6 (0.4-0.9)	.04	2.1 (1.1-3.8)	.02	0.5 (0.4-0.8)	<.001	1.4 (0.8-2.3)	.25
**Education**									
	Primary or less (<9 years)	1		1		1		1	
	Secondary (9-12 years)	2.5 (1.5-4.1)	<.001	2.3 (1.3-4.0)	<.001	1.7 (1.1-2.6)	.02	1.5 (0.9-2.5)	.09
	Postsecondary (>12 years)	6.4 (4.3-9.3)	<.001	2.0 (1.2-3.5)	.01	4.0 (2.8-5.6)	<.001	1.4 (0.8-2.3)	.19
**Occupation**									
	Staff of government institution	1		1		1		1	
	Staff of business or service industry	0.1 (0.1-0.2)	<.001	0.3 (0.2-0.7)	<.001	0.3 (0.2-0.4)	<.001	0.8 (0.5-1.3)	.30
	Farmer	0.1 (0-0.1)	<.001	0.4 (0.2-0.9)	.02	0.3 (0.1-0.4)	<.001	1.3 (0.6-2.6)	.51
	Other (eg, retired, housewife or househusband, and unemployed)	0.2 (0.1-0.2)	.04	0.4 (0.2-0.8)	.01	0.3 (0.2-0.4)	<.001	0.7 (0.5-1.2)	.22
**Source of participants**									
	Community	1		1		1		1	
	Internet	9.6 (6.3-14.5)	<.001	5.0 (3.0-8.0)	<.001	6.9 (5.0-9.5)	<.001	5.5 (3.5-8.4)	<.001

^a^OR: odds ratio.

^b^AOR: adjusted odds ratio.

### Association Between Sociodemographic Variables and KAP Regarding Zika

Univariate and multivariate logistic regression analyses were conducted to explore the association between sociodemographic variables and KAP variables. As shown in Tables 3 and 4, after controlling for other sociodemographic variables, age, education, occupation, and source of participants were associated with knowledge about Zika transmission routes and the association between pregnant women with Zika and newborn babies with microcephaly. Age and source of participants were associated with knowledge that pregnant women are a high-risk group for severe complications. Education and source of participants were associated with knowing that mosquitoes are extremely common in dark, wet places.

Participants aged ≥50 years were more likely to know the transmission route for Zika (OR 2.1, 95% CI 1.1-3.8) and to know of the association between pregnant women with Zika and newborn babies with microcephaly (OR 1.9, 95% CI 1.2-3.1) than participants <30 years old.

The likelihood of knowing the transmission route for Zika (OR 2.0, 95% CI 1.2-3.5) and the association between pregnant women with Zika and newborn babies with microcephaly (OR 2.0, 95% CI 1.3-3.2) were significantly higher among participants with postsecondary education than those with primary or less education.

Participants from nongovernment institutions (staff of businesses or service industries) (OR 0.3, 95% CI 0.2–0.7) and farmers (OR 0.4, 95% CI 0.2-0.9) were less likely to know the transmission route for Zika than participants working in government institutions. Participants recruited on the Internet were more likely to know the transmission route for Zika (OR 5.0, 95% CI 3.0-8.0) and know of the association between pregnant women with Zika and newborn babies with microcephaly (OR 2.1, 95% CI 1.4-3.0) than community-recruited participants.

Knowledge that pregnant women are the main at-risk population for Zika, was more common among participants aged 30-49 years than participants aged under <30 years (OR 1.6, 95% CI 1.0-2.3) and also among Internet-recruited participants compared with community-recruited participants (OR 5.5, 95% CI 3.5-8.4).

**Table 4:** Unadjusted and adjusted (multivariate logistic regression, adjusted for the other factors shown in the table) odds ratios (OR) and 95% CI for knowledge about Zika by sociodemographic variables.

Predictors		Pregnant women infected with Zika might have newborn babies with microcephaly				Mosquitoes are extremely common in dark, wet places			
		OR^a^ (95% CI)	*P* value	AOR^b^ (95% CI)	*P* value	OR (95% CI)	*P* value	AOR (95% CI)	*P* value
**Sex**									
	Female	1		1		1		1	
	Male	1.1 (0.9-1.5)	.34	1.2 (0.9-1.7)	.20	1.2 (0.3-3.9)	.81	1.0 (0.3-3.5)	.99
**Age (years)**									
	<30	1		1		1		1	
	30-49	1.0 (0.8-1.5)	.71	1.1 (0.8-1.6)	.47	0.8 (0.2-3.8)	.84	0.9 (0.2-4.4)	.90
	≥50	0.7 (0.5-1.1)	.10	1.9 (1.2-3.1)	.01	0.5 (0.1-3.0)	.41	0.2 (0.0-1.8)	.16
**Education**									
	Primary or less (<9 years)	1		1		1		1	
	Secondary (9-12 years)	1.7 (1.1-2.6)	.01	1.8 (1.1-2.9)	.02	2.3 (0.2-22.0)	.52	7.8 (0.6-9.5)	.11
	Postsecondary (>12 years)	4.4 (3.2-6.2)	<.001	2.0 (1.3-3.2)	<.001	1.3 (0.3-5.0)	.85	9.8 (1.2-83.5)	.04
**Occupation**									
	Staff of government institution	1		1		1		1	
	Staff of business or service industry	0.1 (0.1-0.2)	<.001	0.3 (0.2-0.4)	<.001	0.9 (0.2-3.8)	.96	0.7 (0.1-3.6)	.65
	Farmer	0.1 (0.1-0.2)	<.001	0.4 (0.2-0.7)	<.001	>999 (<0.01->999)	.98	>999 (<0.01->999)	.96
	Others (eg, retired, housewife or househusband, and unemployed)	0.2 (0.2-0.3)	<.001	0.4 (0.3-0.6)	<.001	2.0 (0.4-9.7)	.39	2.5 (0.3-18.9)	.37
**Source of participants**									
	Community	1		1		1		1	
	Internet	4.3 (3.3-5.6)	<.001	2.1 (1.4-3.0)	0. 001	0.3 (0.1-1.3)	.10	0.1 (0.0-0.5)	.01

^a^OR: odds ratio.

^b^AOR: adjusted odds ratio.

**Table 5:** Unadjusted and adjusted (multivariate logistic regression, adjusted for the other factors shown in the table) odds ratios (OR) and 95% CI for attitudes regarding Zika by sociodemographic variables.

Predictors		I am worried about contracting Zika				I think contracting Zika will cause serious damage to my body			
		OR^a^ (95% CI)	*P* value	AOR^b^ (95% CI)	*P* value	OR (95% CI)	*P* value	AOR (95% CI)	*P* value
**Sex**									
	Female	1		1		1		1	
	Male	1.0 (0.8-1.3)	.97	1.0 (0.8-1.3)	.90	2.0 (1.5-2.7)	<.001	2.1 (1.5-2.8)	<.001
**Age (years)**									
	<30	1		1		1		1	
	30-49	1.0 (0.8-1.4)	.82	1.0 (0.7-1.3)	.86	0.8 (0.6-1.1)	.42	0.9 (0.6-1.2)	.38
	≥50	2.3 (1.5-3.4)	<.001	1.4 (0.9-2.1)	.20	0. 8 (0.5-1.2)	.60	0.9 (0.6-1.5)	.71
**Education**									
	Primary or less (<9 years)	1		1		1		1	
	Secondary (9-12 years)	0.6 (0.4-0.9)	.02	0.7 (0.4-1.1)	.12	1.3 (0.9-2.1)	.19	1.5 (0.9-2.4)	.14
	Postsecondary (>12 years)	0.4 (0.3-0.5)	<.001	0.6 (0.3-0.9)	.01	1.1 (0.8-1.5)	.69	1.5 (0.9-2.4)	.12
**Occupation**									
	Staff of government institution	1		1		1		1	
	Staff of business or service industry	1.7 (1.2-2.4)	<.001	1.1 (0.7-1.6)	.61	1.4 (1.0-1.9)	.049	1.4 (0.9-2.2)	.08
	Farmer	2.9 (1.7-5.1)	.01	1.0 (0.5-2.1)	.90	1.3 (0.8-2.2)	.36	1.6 (0.8-3.2)	.16
	Others (eg, retired, housewife or househusband, and unemployed)	1.5 (1.1-2.1)	<.001	0.9 (0.6-1.3)	.55	1.1 (0.8-1.5)	.53	1.1 (0.7-1.6)	.73
**Source of participants**									
	Community	1		1		1		1	
	Internet	0.4 (0.3-0.6)	<.001	0.6 (0.4-0.9)	.01	0.9 (0.7-1.1)	.30	0.9 (0.6-1.3)	.44

^a^OR: odds ratio.

^b^AOR: adjusted odds ratio.

Participants with postsecondary education were more likely than those with less education to know that mosquitoes were common in dark, wet places (OR 9.8, 95% CI 1.2-83.5); Internet-recruited participants were less likely to know this than community-recruited participants (OR 0.1, 95% CI 0.0-0.5).

Table 5 shows the association between sociodemographic variables and attitude variables. After controlling for other factors, education and sources of participants were associated with worrying about contracting Zika. Participants with postsecondary education were less likely to worry about contracting Zika than participants with primary or less education (OR 0.6, 95% CI 0.3-0.9). Internet-recruited participants were also less likely to worry than community-recruited participants (OR 0.6, 95% CI 0.4-0.9). Male participants were much more likely to think that contracting Zika would cause serious damage (OR 2.1, 95% CI 1.5-2.8).

Table 6 shows the association between sociodemographic variables and practice variables. After controlling for other factors, only the source of participants was found to be associated with the practice of actively seeking information regarding Zika. Internet-recruited participants were more likely to seek information regarding Zika than community-recruited participants (OR 3.3, 95% CI 2.0-5.6).

### Time and Cost

For the paper-based surveys, we spent almost 2 weeks on data collection. For each questionnaire, the investigator got paid 25 RMB, and the respondent received a gift worth 20 RMB. Thus, for 447 questionnaires, the total expenditure for paper-based investigation was 20,115 RMB. Most respondents to the Internet-based survey (83.4%, 518/621) completed the questionnaire in the first week. From days 1 to 7, the number of responses were 327 (52.7%), 107 (17.2%), 26 (4.2%), 58 (9.3%), 30 (4.8%), 7 (1.1%), and 36 (5.8%), respectively. Each online participant got 20 RMB cell phone recharge worth 20 RMB. For the 621 online respondents, the total cost was 12,420 RMB.

**Table 6:** Unadjusted and adjusted (multivariate logistic regression, adjusted for the other factors shown in the table) odds ratios (OR) and 95% CI of practices about Zika by sociodemographic variables.

Predictors		Carry out strategies to prevent mosquito bites at home				Actively seek information about Zika			
		OR^a^ (95% CI)	*P* value	AOR^b^ (95% CI)	*P* value	OR (95% CI)	*P* value	AOR (95% CI)	*P* value
**Sex**									
	Female	1		1		1		1	
	Male	1.1 (0.4-3.1)	.92	1.0 (0.4-3.1)	.94	0.9 (0.6-1.3)	.48	0.9 (0.6-1.3)	.56
**Age (years)**									
	<30	1		1		1		1	
	30-49	1.4 (0.4-4.6)	.34	1.4 (0.4-5.0)	.57	1.3 (0.9-2.1)	.18	1.5 (0.9-2.3)	.11
	≥50	0.8 (0.2-3.0)	.44	1.1 (0.2-5.8)	.89	0.5 (0.3-0.8)	<.001	1.1 (0.6-2.0)	.72
**Education**									
	Primary or less (<9 years)	1		1		1		1	
	Secondary (9-12 years)	1.9 (0.4-9.9)	.69	1.6 (0.3-9.6)	.60	1.3 (0.8-2.1)	.27	1.1 (0.7-1.9)	.67
	Postsecondary (>12 years)	2.0 (0.7-6.0)	.50	1.0 (0.2-4.8)	.97	4.2 (2.8-6.2)	<.001	1.6 (0.9-2.7)	.10
**Occupation**									
	Staff of government institutions	1		1		1		1	
	Staff of business or service industry	0.5 (0.1-1.9)	.86	0.7 (0.1-3.5)	.84	0.2 (0.1-0.4)	<.001	0.6 (0.3-1.1)	.08
	Farmer	0.3 (0.1-1.7)	.35	0.6 (0.1-5.4)	.67	0.2 (0.1-0.4)	<.001	0.8 (0.3-1.8)	.55
	Other (eg, retired, housewife or househusband, and unemployed)	0.6 (0.2-1.9)	.96	0.9 (0.2-4.1)	.65	0.2 (0.2-0.4)	<.001	0.7 (0.4-1.2)	.15
**Source of participants**									
	Community	1		1		1		1	
	Internet	2.4 (0.9-6.6)	.09	2.1 (0.5-8.6)	.30	5.6 (3.8-8.2)	<.001	3.3 (2.0-5.6)	<.001

^a^OR: odds ratio.

^b^AOR: adjusted odds ratio.

## Discussion

### Principal Findings

Rapid assessment of health education needs is necessary for emergent infectious diseases. Usually, paper-based investigations were used. Our study used both paper- and Internet-based approaches to carry out a rapid assessment of health education needs when Zika virus cases were detected in China. This is the first study in the Zhejiang Province that has used both the methods to investigate a public health issue and provides a detailed comparison between the two. The main finding of our study was that participants in the Internet-based survey had a higher level of basic knowledge and more positive attitudes and behaviors toward Zika than participants in the paper-based survey, even after controlling for factors such as age, sex, and education level. The Internet-based survey required fewer resources and was much faster than the paper-based survey. However, if Internet-based surveys are used to carry out emergency assessments of health education needs, we should be cautious about the results because self-selected participants of Internet-based surveys do not represent the general population.

Our study found that participants in the Web-based study had characteristics different from those in the paper-based study. First, the Internet-recruited sample was younger, with 95.2% (591/621) of participants <50 years of age, whereas in the community-recruited sample only 67.6% (302/447) were <50 years of age. Second, participants in the Web-based study had higher levels of education than those in the paper-based study. Over 90% (566/621, 91.1%) of the Internet-recruited participants had postsecondary education compared with 31.8% (142/447) of community-recruited participants. Third, occupational distribution comparisons also found a significant difference between the two groups. More participants in the Web-based study were employed in government agencies or businesses, whereas in the paper-based study there were more farmers and people who stayed at home (eg, retired, housewife or househusband, and unemployed). This correlates with education levels of the two groups. Other studies have also shown that participants in Web-based studies have characteristics different from those participating in paper-based studies [14-17]. Due to the different characteristics of Internet users and other community residents, different health education methods should be applied. For example, for the elderly and people with low levels of education, materials such as posters, bulletin boards, banners, and booklets would be more helpful. For younger and well-educated people who are used to obtaining information via the Internet, Web-based health education using new media such as WeChat, blogs, and Web pages may be more appropriate.

This study found no difference in sex distribution between the two groups. However, both groups had more female than male participants (about 7:3). This might be because women are more concerned about their health than men [18-20], so they are more willing to participate in investigations on health-related issues.

By comparing the cost and time of the paper- and Internet-based surveys, we found that our Internet-based study was faster and more cost-effective. In this study, over 500 questionnaires were collected online within 1 week, whereas it took 2 weeks to collect 447 questionnaires through face-to-face interviews. Additionally, the Web-based study only cost half as much as the paper-based investigation.

Under conditions of new infectious diseases, the ability to rapidly assess health education needs is necessary for health workers to understand the public’s KAP about the new disease to carry out targeted health education and risk communication. Our study confirmed that, compared with a paper-based survey, collecting data with a Web-based survey involves considerably fewer resources including money, time, and human resources [21-23], which is what health education assessment needs for an emerging infectious disease. However, participants in the Internet-based survey might not represent the whole population; thus, if a Web-based survey was used to carry out an emergency assessment of health education needs, we should be quite cautious about the results.

The study also found that participants in the Internet-based survey had a higher level of knowledge about Zika than participants in the paper-based survey. This might be because of having more young and well-educated participants in the Web-based survey. However, after controlling for sociodemographic variables such as age, education, and sex, Internet participants in the Internet-based survey still had a higher level of knowledge about Zika than participants in the paper-based survey. As new media such as WeChat and microblogs become a primary source of information, health workers move from community-based to Internet-based health education, there is a risk of health inequity. Our study suggests that in the event of an outbreak of a new infectious disease, while providing health information online, the government, health workers, and the media should also ensure sufficient access to health information for older and less well-educated people in the community to ensure equity.

Fewer participants in the paper-based survey worried about contracting Zika than participants in the Web-based survey. This might be because they knew less about Zika, and ignorance gave them less anxiety. Our study found that 98.50% (1052/1068) of participants carried out strategies to prevent mosquito bites at home. This suggests that the public of Zhejiang have positive behaviors toward preventing mosquito-borne diseases. Participants in the Internet-based survey tended to seek information on Zika more actively than participants in the paper-based survey. This suggests that, in an outbreak of a new infectious disease, to achieve better health education, health workers should provide information in a range of ways, which will be attractive to the community, to ensure positive messages are heard by the public. Web-based health information should be communicated through official channels as much as possible, to minimize the flow of inaccurate information and rumors.

### Limitations

This study has some limitations. First, selection bias might exist. Data for the paper-based study were collected in the community during the daytime, so participation may have been biased toward the elderly and unemployed. Online participants were usually younger and better educated and also had more interest in the study. Thus, KAP results about Zika from both our paper- and Internet-based surveys may not be generalizable to the whole population. Second, to get a quick understanding of the levels of KAP about Zika, we kept the questionnaire short, which limited the depth of the study.

### Conclusions

Our study provides valuable insights into KAP related to Zika among people in the southern part of China. The Internet-based survey had a larger proportion of participants with a basic knowledge regarding Zika, and they tended to seek information on Zika more actively and be less worried about contracting Zika than participants in the paper-based survey. Although, the Internet-based survey involved fewer resources and was much faster than the paper-based survey in the community, if only an Internet-based survey were used to carry out an emergency assessment of health education needs, you would need to be cautious about the generalizability of the results. Finally, our study suggests that in the outbreak of a new infectious disease, as well as providing health information on the Internet, the government and health workers should also ensure adequate access to health information for older and less educated people in the community to achieve greater equity in health.

## Abbreviations

AOR: adjusted odds ratio
OR: odds ratio
KAP: knowledge, attitudes, and practices

## References

[1] Dick GW, Kitchen SF, Haddow AJ (1952). Zika virus. I. Isolations and serological specificity. Trans R Soc Trop Med Hyg.

[2] Ioos S, Mallet HP, Leparc Goffart I, Gauthier V, Cardoso T, Herida M (2014). Current Zika virus epidemiology and recent epidemics. Med Mal Infect.

[3] Staples JE, Dziuban EJ, Fischer M, Cragan JD, Rasmussen SA, Cannon MJ, Frey MT, Renquist CM, Lanciotti RS, Muñoz JL, Powers AM, Honein MA, Moore CA (2016). Interim guidelines for the evaluation and testing of infants with possible congenital Zika virus infection - United States, 2016. MMWR Morb Mortal Wkly Rep.

[4] Baidu (2017). Baike.baidu.

[5] Wikipedia.

[6] Guo S, Ling F, Hou J, Wang J, Fu G, Gong Z (2014). Mosquito surveillance revealed lagged effects of mosquito abundance on mosquito-borne disease transmission: a retrospective study in Zhejiang, China. PLoS One.

[7] SteelFisher GK, Blendon RJ, Bekheit MM, Lubell K (2010). The public's response to the 2009 H1N1 influenza pandemic. N Engl J Med.

[8] Blendon RJ, Benson JM, DesRoches CM, Raleigh E, Taylor-Clark K (2004). The public's response to severe acute respiratory syndrome in Toronto and the United States. Clin Infect Dis.

[9] Hewitt-Taylor J (2014). Using the Internet as a source of information and support: a discussion paper on the risks and benefits for children and young people with long-term conditions. J Innov Health Inform.

[10] van Gelder MM, Schouten NP, Merkus PJ, Verhaak CM, Roeleveld N, Roukema J (2015). Using web-based questionnaires and obstetric records to assess general health characteristics among pregnant women: a validation study. J Med Internet Res.

[11] Ekman A, Dickman PW, Klint A, Weiderpass E, Litton JE (2006). Feasibility of using web-based questionnaires in large population-based epidemiological studies. Eur J Epidemiol.

[12] Mao C, Wu XY, Fu XH, Di MY, Yu YY, Yuan JQ, Yang ZY, Tang JL (2014). An internet-based epidemiological investigation of the outbreak of H7N9 Avian influenza A in China since early 2013. J Med Internet Res.

[13] Gu H, Chen B, Zhu H, Jiang T, Wang X, Chen L, Jiang Z, Zheng D, Jiang J (2014). Importance of Internet surveillance in public health emergency control and prevention: evidence from a digital epidemiologic study during avian influenza A H7N9 outbreaks. J Med Internet Res.

[14] Hirsch O, Löltgen K, Becker A (2014). Comparing health survey data from Internet- and paper-based convenience samples of lesbian women in Germany. Sex Health.

[15] Gosling SD, Vazire S, Srivastava S, John OP (2004). Should we trust web-based studies? A comparative analysis of six preconceptions about internet questionnaires. Am Psychol.

[16] Engan HK, Hilmarsen C, Sittlinger S, Sandmæl JA, Skanke F, Oldervoll LM (2016). Are web-based questionnaires accepted in patients attending rehabilitation?. Disabil Rehabil.

[17] Hirsch O, Hauschild F, Schmidt MH, Baum E, Christiansen H (2013). Comparison of Web-based and paper-based administration of ADHD questionnaires for adults. J Med Internet Res.

[18] Hadjimina E, Furnham A (2017). Influence of age and gender on mental health literacy of anxiety disorders. Psychiatry Res.

[19] Hernandez EM, Margolis R, Hummer RA (2016). Educational and gender differences in health behavior changes after a gateway diagnosis. J Aging Health.

[20] Gu H, Jiang Z, Chen B, Zhang JM, Wang Z, Wang X, Cai J, Chen Y, Zheng D, Jiang J (2015). Knowledge, attitudes, and practices regarding avian influenza A (H7N9) among mobile phone users: a survey in Zhejiang Province, China. JMIR Mhealth Uhealth.

[21] Heiervang E, Goodman R (2011). Advantages and limitations of web-based surveys: evidence from a child mental health survey. Soc Psychiatry Psychiatr Epidemiol.

[22] Weber BA, Yarandi H, Rowe MA, Weber JP (2005). A comparison study: paper-based versus web-based data collection and management. Appl Nurs Res.

[23] Uhlig CE, Seitz B, Eter N, Promesberger J, Busse H (2014). Efficiencies of Internet-based digital and paper-based scientific surveys and the estimated costs and time for different-sized cohorts. PLoS One.

